# Alcohol and tobacco use among sexual and gender minority cancer survivors in relation to urbanicity/rurality

**DOI:** 10.1007/s10552-025-02065-5

**Published:** 2025-09-09

**Authors:** Tyra Robertson, James L. Fisher, Joanne G. Patterson, N. F. N. Scout, Elizabeth K. Arthur

**Affiliations:** 1 Department of Nursing Research, The Ohio State University Comprehensive Cancer Center Arthur G. James Cancer Hospital and Richard J. Solove Research Institute, 460 W 10th Ave, Columbus, OH 43210 USA; 2 The Arthur G. James Cancer Hospital and Richard J. Solove Research Institute, Columbus, OH USA; 3 College of Public Health, The Ohio State University, Columbus, OH USA; 4 National LGBT Cancer Network, Providence, RI USA

**Keywords:** Urban, Rural, Sexual and Gender Minorities, Cancer, Tobacco, Alcohol

## Abstract

**Purpose:**

Understanding how place of residence affects cancer-related health risks is paramount to addressing health disparities in sexual and gender minority (SGM) cancer survivors. This study examined the associations between urbanicity and other social drivers of health on current tobacco and alcohol use in SGM cancer survivors.

**Methods:**

The OUT: National Cancer Survey Study was a cross-sectional, online survey created by the National LGBT Cancer Network (NLCN) from September 2020 to March 2021, targeting U.S. adults identifying as SGM and previously diagnosed with cancer. We examined associations between self-described residential area (urban, suburban, and rural) and other social drivers of health and tobacco and alcohol use.

**Results:**

Of *n* = 2,371 participants, *n* = 350 reported tobacco use and *n* = 359 reported ≥ 2 alcoholic drinks/day. The odds of consuming ≥ 2 alcoholic drinks/day were lower among those living in suburban (vs urban) areas (adjusted odds ratio [AOR] = 0.74; 95% CI: 0.56–0.96) and those reporting a disability (AOR = 0.62; 95% CI: 0.46–0.83) and were higher among Black/African American (versus White) cancer survivors (AOR = 2.36; 95% CI: 1.32–4.22). The odds of current tobacco use did not differ significantly based on place of residence, but decreased with increasing age (AOR = 0.97; 95% CI = 0.96–0.98), were lower for those with graduate school education (AOR = 0.29; 95% CI = 0.16–0.54), and health insurance (AOR = 0.30; 95% CI = 0.16–0.59), and were greater among Black/African American (versus White) (AOR = 2.55; 95% CI: 1.36–4.80) and Hispanic (versus non-Hispanic) (AOR = 1.77; 95% CI = 1.04–3.00) SGM cancer survivors.

**Conclusion:**

Urbanicity/Rurality was significantly associated with alcohol use among SGM cancer survivors. Social drivers of health are crucial factors for researchers and clinicians intervening to improve the health of SGM cancer survivors.

**Supplementary Information:**

The online version contains supplementary material available at 10.1007/s10552-025-02065-5.

## Background

Approximately 7.6% of the U.S. population identifies as sexual or gender minority (SGM), including lesbian, gay, bisexual, transgender, queer or other diverse genders and sexual orientations (LGBTQ+) [1]. In 2025, it is projected there will be over 2 million new cancer cases and over 600,000 cancer deaths in the United States [2, 3]. By extrapolation, there will be an estimated 142,000 SGM individuals among the newly diagnosed. This is likely an under-estimation because SGM people report more risk factors for cancer than their cisgender, heterosexual counterparts including tobacco and alcohol use, nulliparity (never given birth), and obesity [4, 5]. Emerging evidence supports higher rates of some cancers in specific subpopulations of SGM people [6, 7]. Equally important, many SGM cancer survivors in the U.S. experience unmet needs related to diagnosis and treatment [8, 9], increased risk for cancer recurrence [10], and are at higher risk for some comorbidities [11–13].

Tobacco and alcohol use are major risk factors for de novo and recurrent cancer and other health comorbidities. In fact, tobacco use is the leading cause of preventable death [14]. In 2019, cigarette smoking was the leading risk factor contributing to cancer cases and deaths overall (19.3% and 28.5%, respectively), followed by excess body weight (7.6% and 7.3%, respectively), and alcohol use (5.4% and 4.1%, respectively) [15]. Tobacco use is associated with cancer of at least 12 sites, heart disease, stroke, lung diseases, diabetes, and chronic obstructive pulmonary disease, which includes emphysema and chronic bronchitis [15, 16]. Furthermore, higher levels of alcohol use are associated with increased risk of cancer of at least seven sites/types, as well as high blood pressure, heart disease, stroke, liver disease, and digestive problems [15–17].

Unfortunately, SGM people have higher rates of alcohol and tobacco use [18–20]. For example, smoking rates among sexual minority women are almost three times higher than those of women in the general population [21, 22], and rates of high-intensity and binge drinking are approximately twice those of heterosexual women [23]. Among sexual minority men, odds ratios for smoking range between 2.0 and 2.5 indicating higher odds of smoking when compared to heterosexual men [19]. Transgender and gender diverse individuals are 2–3 times more likely to report current tobacco use than cisgender individuals, even after adjustment for potential confounders [24].

Higher rates of substance use in SGM people are rooted in the historical context of SGM communities. Repressive anti-gay legislation forced SGM culture to become secretive and evasive to escape discrimination and violence [25], as such clubs and bars became a common place to meet and socialize [26]. The 1970s saw the establishment of urban gay clubs, and Absolut® vodka heralded a new era of SGM marketing when they began advertising in the SGM press [27]. Targeted marketing to SGM people further expanded when leading tobacco manufacturer RJ Reynolds® identified parts of SGM communities as key target markets [28]. SGM communities have demonstrated considerable resilience in the face of persistent historical discrimination and prejudice [29, 30]. While advancements such as marriage equality, decriminalization, healthcare protections, and increased media representation mark significant progress, substantial challenges remain in achieving full equity in civil rights. Since 2020, there has been a steep rise in SGM discriminatory policies introduced and passed in state legislatures across the U.S. [31], and emerging evidence suggests that greater discrimination is associated with increase odds of smoking, heavy drinking, and binge drinking among SGM adults [32].

The historical and contemporary contexts in which SGM people live contribute to minority stress, defined as additional stress experienced due to one’s minoritized sexual orientation or gender identity, and challenges with social drivers of health which may exacerbate disparities [33–35]. For example, sexual minority women exhibit substantial disparities in drivers of tobacco use, including economic indicators (*e.g.,* unemployment, means-tested assistance), health insurance, food insecurity, exposure to tobacco marketing, and fewer perceived deterrents to smoking [21, 36–38]. Further, there is evidence of a significant relationship between smoking, binge drinking, and several other social drivers of health, including employment status, education, and income in SGM people [39].

Of particular interest is the social driver of health defined by area of residence, shown to influence both rates of tobacco and alcohol use as well as rates of specific cancers. In the U.S., there is clear spatial patterning of SGM people and same-sex couples in urban and wealthier areas [40, 41]; however, there is limited information about neighborhood-level residential differences between SGM and non-SGM people [41–43]. Although spatial patterning of SGM is likely more nuanced than that witnessed as an exclusively population density-based phenomenon, urban/suburban/rural) residence in the U.S. can influence health outcomes due to the different socioeconomic and environmental stressors experienced by residents [44]. Compared to urban residents, rural residents (estimated 14–19% of the U.S. population) have higher poverty rates, lower educational attainment, lack of transportation, and lack of healthcare services [45]. Rural residents are also more likely to be uninsured and often travel farther to access care, decreasing use of safety net providers [46, 47]. In the U.S., rural residents tend to be older, have lower adherence to preventive care, and engage in risky health behaviors more often compared to those living in urban and suburban areas [48, 49]. Consequently, they are at a higher risk of developing chronic diseases, including cancer of many sites/types [50].

Cigarette smoking is more prevalent in rural versus urban areas, and the trend in cigarette use is declining more rapidly among urban than rural populations [51]. Findings from a systematic review concerning rural–urban comparisons of alcohol use suggests, in the United States, higher prevalences of high-risk alcohol use and alcohol-related harm in rural versus urban areas [52]. However, findings are mixed, such that urban (versus rural) locations also demonstrate higher prevalence of excessive alcohol use, while prevalence of abstinence is lowest in suburban areas [52, 53].

Cancer incidence and mortality rates differ in rural, urban, and suburban areas [14, 51]. Among cancers overrepresented among SGM people, lung and colorectal cancer are more prevalent in rural populations [54]. The higher incidence of lung cancer in rural areas is largely attributable to higher prevalence of tobacco use [14]. In contrast, breast and prostate cancer incidence rates are higher in urban areas [54].

Research has described risk factors (*e.g.,* cultural mores, discrimination, and socioeconomic status) and health outcomes among SGM people in rural and urban areas [55]. but few have *compared* risks or outcomes by place of residence, particularly for those previously diagnosed with cancer. Therefore, little is known about the tobacco and alcohol use patterns of SGM cancer survivors. However, SGM cancer survivors who continue using tobacco and alcohol continue to be at risk for recurrence, second cancers, and other comorbidities. We aimed to explore tobacco and alcohol use as health risk behaviors after cancer diagnosis among urban, suburban, and rural SGM cancer survivors. Our results can inform tailored risk reduction treatment and survivorship care for SGM people with cancer.

## Methods

### Research design

This secondary analysis was completed using the *OUT: National Cancer Survey Study.* (hereafter, referred to as the OUT Study). The OUT Study is a cross-sectional, online survey created by the National LGBT Cancer Network (NLCN) to understand the needs of SGM cancer survivors to identify opportunities for creating SGM affirming environments in the cancer context. The OUT Study recorded experiences such as cancer diagnosis and treatment, disability status, social networks, support, cancer impact, insurance status, cancer screenings, and health behavior risk factors. Participants were recruited from NLCN’s partner organizations, specifically those working closely with LGBTQI + cancer survivors, social media, and paid advertisement; a total of 3,750 SGM cancer survivors were recruited. Individuals were eligible to participate if they self-identified as SGM adults living in the US with previously diagnosed cancer. Specifically, potential participants were asked: “Do you self-identify as LGBTQI + ?” (Note that it was possible for a participant to respond “Yes” to this question, then to describe their sexual orientation as “Straight.”) Participants were provided with a downloadable, informed consent document and asked to read it prior to starting the online survey. The survey was available to participants from September 2020 to March 2021 in both English and Spanish [56]. To evaluate responses for the presence of bots, survey responses were assessed for congruence of answers and metrics such as IP addresses, time of response, and email addresses, if provided, were reviewed any concerning responses were removed from the final data set.

### Alcohol use

Alcohol use was defined by the question: “On average, how many alcoholic drinks do you drink on an average day?” The parameters were, “One drink is equivalent to a 12-oz beer, a 5-oz glass of wine, or a drink with one shot of liquor.” Respondents selected from none to five or more drinks per day. Responses were dichotomized into: ‘fewer than two drinks per day’ and ‘two or more drinks per day’ [57].

### Tobacco use

Tobacco use was defined by the question: “Do you currently use any of the following tobacco products?” Respondents selected all that applied. The options were, “Cigarettes,” “Cigars or Cigarillos,” “Electronic cigarettes, vapes, or JUUL,” “Hookah,” “Chewing Tobacco, Snus, or Snuff,” “I do not currently use tobacco products,” “Don’t know/ Prefer not to answer.” Responses were dichotomized into: ‘current tobacco use’ and ‘no current tobacco use.’

### Urbanicity/rurality

The primary independent variable, urbanicity/rurality, was collected using the question: “How would you describe where you currently live?” Participants subjectively described their residence as one of the following options: “Urban (e.g., major capital city),” “Suburban (*e.g.,* town or city outside a major capital city),” “Rural or Remote,” and “I prefer not to share this information.” We use the term urbanicity/rurality instead of ‘rural–urban classification’ to avoid confusing this self-report or perceived area of residence to a formal variable based on zip code, census tract, or city size.

### Covariates

Socio-demographic information was self-reported by participants. The full list of sociodemographic variables with response options is reported in Table 2. Respondents who reported their sexual orientation as bisexual or pansexual were grouped together. Due to small number of responses, participants who reported sexual orientations of asexual, straight, multiple orientations, additional orientations, and those who preferred not to answer were grouped together. Respondents who reported one race and whose race was anything other than White/European or Black/African American were grouped as Indigenous, & people of color (IPOC). Those describing their race using additional descriptions (*e.g.,* ‘human’), which were not clearly aligned with other categories, could not be classified and were included with those who preferred not to share. Respondents who reported their gender identity as transgender, genderqueer, gender non-conforming, non-binary, and additional gender identities were grouped together.

### Statistical analysis

Descriptive statistics were used to describe distributions of selected demographic characteristics and alcohol and tobacco use. Univariate (unadjusted) logistic regression was used to obtain odds ratios (OR) and 95% confidence intervals (CI) reflecting associations between urbanicity/rurality (urban/suburban/rural) and high-risk health behaviors (current tobacco use, and ≥ 2 alcoholic drinks/day (hereafter, referred to as heavy drinking). Additional potential confounders (age, gender identity, sex assigned at birth, sexual orientation, race, ethnicity, education, insurance and insurance types, and years since diagnosis) were also examined for associations with high-risk health behaviors using univariate (unadjusted) logistic regression. Missing information concerning covariates was not interpolated or estimated using regression modeling; missing information was not included in regressions. Odds ratios were characterized as differences in the odds of high-risk health behaviors according to levels of selected characteristics. The final multivariable (adjusted) model was built to include all potentially confounding factors. For all analyses, p-values were 2-sided, with the alpha set at 0.05 for all hypothesis tests. Statistical analyses were conducted using SPSS version 28.0.1.1.

## Results

### Sample characteristics

The OUT Study included responses from 4,517 participants. After excluding those not completing the survey, 2,519 participants remained. After further excluding those not meeting the inclusion criteria and those without usable responses to questions about current tobacco use or current alcohol use, 2,440 participants remained. Of these, 69 did not report usable responses concerning urban/rural/suburban place of residence, leaving 2,372 participants. Among these 2,372 participants, the most common cancer types were prostate, breast, skin cancer-basal or squamous, and colorectal (Table 1). Participants may have selected multiple cancer types; therefore, the sums of frequencies of cancer types exceed the sample sizes.

**Table 1:** Self-reported cancer types among 2,372 participants

Cancer Type	N
Anal	152
Bladder	74
Breast	414
Cervical	56
Colorectal	195
Head and Neck	125
Hodgkin’s Lymphoma	71
Kidney	73
Leukemia	79
Liver	29
Lung	118
Non-Hodgkin Lymphoma	179
Ovarian	76
Pancreatic	29
Prostate	445
Skin Cancer – Melanoma	142
Skin Cancer – Basal or Squamous Cell	202
Thyroid	79
Other	466
Don’t Know/Prefer Not to Answer	5

Note that column does not total to sample sizes due to participant reports of multiple cancers

There were 359 participants (15.1%) who reported consuming ≥ 2 alcoholic drinks/day. This level of consumption was significantly more common among urban participants (18.3%) compared with suburban (13.7%) and rural (13.0%) participants.

There were 350 participants (14.8%) who reported current use of at least one tobacco product. The percentage of current tobacco users was higher in rural areas (17.6%) compared to suburban and urban areas, 13.8% and 15.6%, respectively. Of current tobacco users, 301 reported use of only one product. The majority (*n* = 222) reported cigarette use alone (39.2% urban, 40.5% suburban, 20.3% rural), 49 reported use of electronic cigarettes, vape or JUUL alone (32.7% urban, 51.0% suburban, 16.3% rural), and 24 reported cigars or cigarillos alone (37.5% urban, 50.0% suburban, 12.5% rural). Of the 49 participants reporting use of more than one type of tobacco product, 49.0% were urban, 38.8% were suburban, and 12.2% were rural.

There were 75 participants reporting both current tobacco use and consuming at least 2 alcoholic drinks/day.

Among all participants, the majority were male, gay, White/European American, non-Hispanic, living in a suburban area, and reported no disabilities (Table 2). (Supplemental Table 1 shows the breakdown of sociodemographic factors for the subset of participants from whom we received responses for each variable of interest.)

**Table 2:** Study sample characteristics and associations with heavy alcohol use & tobacco use among sexual and gender minority cancer survivors

Variable Characteristic	Alcohol Use (≥ 2 drinks per day) *n* (Column %)		Tobacco Use *n* (Column %)	
	No	Yes	No	Yes
Age in years (mean SD)				
	58.2 (11.2)	59.8 (11.2)	59.0 (10.9)	55.1 (12.1)
Gender Identity				
Male	1129 (79.8%)	285 (20.2%)	1158 (82.7%)	243 (17.3%)
Female	701 (91.5%)	65 (8.5%)	681 (89.3%)	82 (10.7%)
Transgender, Genderqueer, Gender non-conforming, Non-Binary, Additional Gender Identities	135 (93.8%)	9 (6.3%)	116 (82.9%)	24 (17.1%)
Sex assigned at birth				
Male	1159 (80.1%)	288 (19.9%)	1183 (82.7%)	248 (17.3%)
Female	811 (92.0%)	71 (8.0%)	779 (88.6%)	100 (11.4%)
Sexual Orientation				
Gay	1022 (79.2%)	268 (20.8%)	1064 (83.4%)	212 (16.6%)
Lesbian	521 (92.0%)	45 (8.0%)	506 (89.2%)	61 (10.8%)
Bisexual, Pansexual	141 (89.2%)	17 (10.8%)	127 (80.9%)	30 (19.1%)
Queer	51 (98.1%)	1 (1.9%)	46 (90.2%)	5 (9.8%)
Asexual, Straight, Multiple Orientations–- Additional Orientations	242 (89.6%)	28 (10.4%)	224 (84.2%)	42 (15.8%)
Race				
White	1708 (85.1%)	299 (14.9%)	1703 (85.6%)	287(14.4%)
Black/African American	52 (69.3%)	23 (30.7%)	48 (67.6%)	23 (32.4%)
Indigenous and non-black/African American people of color	31 (91.2%)	3 (8.8%)	31 (86.1%)	5 (13.9%)
Biracial/multiracial	105 (83.3%)	21 (16.7%)	105 (85.4%)	18 (14.6%)
Unclassifiable/Prefer Not to Answer	82 (86.3%)	13 (13.7%)	81 (82.7%)	17 (17.3%)
Ethnicity				
Non-Hispanic	1811 (85.1%)	318 (14.9%)	1806 (85.6%)	303 (14.4%)
Hispanic	104 (80.0%)	26 (20.0%)	95 (73.6%)	34 (26.4%)
Education				
Highschool Diploma or less	75 (82.4%)	16 (17.6%)	65 (71.4%)	91 (28.6%)
Some College/Vocational School	345 (86.5%)	54 (13.5%)	282 (71.4%)	395 (28.6%)
College or Vocational School/Degree Certificate	748 (84.5%)	137 (15.5%)	734 (83.4%)	880 (16.6%)
Grad School	787 (84.5%)	144 (15.5%)	863 (93.7%)	58 (6.3%)
Insurance				
Yes	53 (77.9%)	15 (22.1%)	40 (58.0%)	29 (42.0%)
No	1910 (84.9%)	340 (15.1%)	1914 (85.8%)	316 (14.2%)
Private insurance				
Yes	823 (83.4%)	164 (16.6%)	801 (81.5%)	182 (18.5%)
No	1155 (85.6%)	195 (14.4%)	1167 (87.4%)	168 (12.6%)
Medicaid				
Yes	1739 (84.1%)	328 (15.9%)	1767 (86.0%)	288 (14.0%)
No	239 (88.5%)	31 (11.5%)	201 (76.4%)	62 (23.6%)
Medicare				
Yes	1242 (86.3%)	198 (13.8%)	1220 (84.9%)	217 (15.1%)
No	736 (82.1%)	161 (17.9%)	748 (84.9%)	133 (15.1%)
Disability				
Yes	1172 (82.2%)	253 (17.8%)	1245 (87.4%)	180 (12.6%)
No	713 (88.8%)	90 (11.2%)	631 (80.3%)	155 (19.7%)
Years since Diagnosis				
0–2 years	644 (86.2%)	103 (13.8%)	603 (82.0%)	132 (18.0%)
3–5 years	371 (83.2%)	75 (16.8%)	377 (84.7%)	68 (15.3%)
6–10 years	395 (83.7%)	77 (16.3%)	407 (86.6%)	63 (13.4%)
11 + years	525 (84.5%)	96 (15.5%)	541 (87.7%)	76 (12.3%)
Place of Residence				
Urban	737 (81.7%)	165 (18.3%)	756 (84.4%)	140 (15.6%)
Suburban	933 (86.3%)	148 (13.7%)	921 (86.2%)	148 (13.8%)
Rural	308 (87.0%)	46 (13.0%)	291 (84.9%)	62 (17.6%)

*United States, March 2021*

Percentages exclude missing/no response/unclassifiable data for each variable. Column sum does not match total column sample size due to missing/no response/unclassifiable data

### Univariate associations

There was a statistically significant univariate association between urbanicity/rurality and heavy drinking. Compared to urban residents, odds of heavy drinking were significantly lower among suburban and rural/remote participants (OR = 0.71; 95% CI = 0.56–0.90; OR = 0.67; 95% CI = 0.47–0.95, respectively). In univariate analyses, additional significant associations were identified for age, gender identity, sex assigned at birth, sexual orientation, race, Medicare status, and disability status. Per Table 3, there was no significant association between urbanicity/rurality and current tobacco use. Statistically significant associations with tobacco use were identified within categories of all potential confounders with the exception of having Medicare insurance.

**Table 3:** Univariate (unadjusted) associations between study sample characteristics, heavy alcohol use, and current tobacco use among sexual and gender minority cancer survivors

Variable (Referent)	Alcohol Use (≥ 2 drinks per day)		Tobacco Use	
	OR (95% CI)	p-value	OR (95% CI)	p-value
Age in years (mean SD)				
	1.01 (1.00–1.02)	0.013*	0.97 (0.96–0.98)	< 0.001*
Gender Identity (ref: male)				
Female	0.37 (0.28–0.49)	< 0.001*	0.57 (0.44–0.75)	< 0.001*
Transgender, Genderqueer, Gender non-conforming, Non-Binary, Additional Gender Identities	0.26 (0.13–0.53)	< 0.001*	0.99 (0.62–1.56)	0.952
Sex assigned at birth (ref: male)				
Female	0.35 (0.27–0.46)	< 0.001*	0.61 (0.48–0.79)	< 0.001*
Sexual Orientation (ref: gay)				
Lesbian	0.33 (0.24–0.46)	< 0.001*	0.61 (0.45–0.82)	0.001*
Bisexual, Pansexual	0.46 (0.27–0.77)	0.003*	1.19 (0.78–1.81)	0.432
Queer	0.08 (0.01–0.54)	0.010*	0.55 (0.21–1.39)	0.204
Asexual, Straight, Multiple Orientations–- Additional Orientations	0.44 (0.29–0.67)	< 0.001*	0.94 (0.66–1.35)	0.741
Race (ref: white)				
Black/African American	2.53 (1.52–4.19)	< 0.001*	2.84 (1.70–4.75)	< 0.001*
Indigenous and non-black/African American people of color	0.55 (0.17–1.82)	0.330	0.96 (0.37–2.48)	0.928
Biracial/multiracial	1.14 (0.70–1.85)	0.590	1.02 (0.61–1.70)	0.948
Unclassifiable/Prefer Not to Answer	0.91 (0.50–1.65)	0.745	1.25 (0.73–2.12)	0.424
Ethnicity (ref: not Hispanic)				
Hispanic	1.42 (0.91–2.22)	0.121	2.13 (1.41–3.21)	< 0.001*
Education (ref: HS diploma or less)				
Some College/Vocational School	0.73 (0.40–1.35)	0.321	1.00 (0.61–1.66)	0.995
College or Vocational School/Degree Certificate	0.86 (0.49–1.52)	0.600	0.50 (0.31–0.81)	0.005*
Graduate School	0.86 (0.49–1.51)	0.596	0.17 (0.10–0.29)	< 0.001*
Insurance (ref: no)				
Yes	0.63 (0.35–1.13)	0.120	0.23 (0.14–0.37)	< 0.001*
Private insurance (ref: no)				
Yes	0.85 (0.68–1.06)	0.151	0.63 (0.50–0.80)	< 0.001*
Medicaid (ref: no)				
Yes	0.69 (0.47–1.02)	0.061	1.90 (1.39–2.58)	< 0.001*
Medicare (ref: no)				
Yes	1.37 (1.09–1.72)	0.006*	1.00 (0.79–1.26)	0.998
Disability (ref: no)				
Yes	0.59 (0.45–0.76)	< 0.001*	1.70 (1.34–2.15)	< 0.001*
Years since Diagnosis (ref: 0–2 years)				
3–5 years	1.26 (0.91–1.75)	0.156	0.82 (0.60–1.13)	0.235
6–10 years	1.22 (0.88–1.68)	0.227	0.71 (0.51–0.98)	0.037*
11 + years	1.14 (0.85–1.55)	0.383	0.64 (0.47–0.87)	0.004*
Place of Residence (ref: urban)				
Suburban	0.71 (0.56–0.90)	0.005*	0.87 (0.68–1.12)	0.267
Rural	0.67 (0.47–0.95)	0.025*	1.15 (0.83–1.60)	0.402

*United States, March 2021*

*Significant values are indicated with an asterisk

### Multivariable analyses

The final multivariable models (Table 4) included all potentially confounding factors. After adjustment for confounding by other factors, odds of heavy drinking among suburban participants were 26% lower than those of urban participants (AOR = 0.74; 95% CI = 0.56–0.96). The odds of heavy drinking after cancer diagnosis were significantly higher among Black/African American (versus White) (AOR = 2.36; 95% CI = 1.32–4.22) participants and significantly lower among those reporting a disability (AOR = 0.62; 95%CI = 0.46–0.83).

**Table 4:** Multivariable (adjusted) associations between study sample characteristics, heavy alcohol use, and current tobacco use among sexual and gender minority cancer survivors

Variable (Referent)	Alcohol Use (≥ 2 drinks per day)		Tobacco Use	
	OR (95% CI)	p-value	OR (95% CI)	p-value
Age in years (mean SD)				
	1.00 (0.98–1.01)	0.352	0.97 (0.96–0.98)	< 0.001*
Gender Identity (ref: male)				
Female	0.69 (0.22–2.17)	0.525	0.54 (0.20–1.48)	0.232
Transgender, Genderqueer, Gender non-conforming, Non-Binary, Additional Gender Identities	0.73 (0.23–2.27)	0.583	0.71 (0.26–1.90)	0.492
Sex assigned at birth (ref: male)				
Female	0.81 (0.28–2.28)	0.683	0.74 (0.30–1.85)	0.515
Sexual Orientation (ref: gay)				
Lesbian	0.59 (0.27–1.30)	0.193	1.55 (0.77–3.13)	0.223
Bisexual, Pansexual	0.63 (0.30–1.34)	0.230	1.31 (0.69–2.48)	0.412
Queer	0.13 (0.02–1.05)	0.055	0.86 (0.29–2.59)	0.789
Asexual, Straight, Multiple Orientations–- Additional Orientations	0.68 (0.37–1.25)	0.212	1.31 (0.75–2.28)	0.349
Race (ref: white)				
Black/African American	2.36 (1.32–4.22)	0.004*	2.55 (1.36–4.80)	0.004*
Indigenous and non-black/African American people of color	0.62 (0.18–2.08)	0.436	1.20 (0.43–3.31)	0.730
Biracial/multiracial	1.27 (0.70–2.30)	0.429	0.65 (0.33–1.28)	0.215
Unclassifiable/Prefer Not to Answer	1.04 (0.45–2.43)	0.922	0.80 (0.36–1.80)	0.593
Ethnicity (ref: not Hispanic)				
Hispanic	1.21 (0.70–2.09)	0.492	1.77 (1.04–3.00)	0.035*
Education (ref: HS diploma or less)				
Some College/Vocational School	0.72 (0.36–1.45)	0.359	1.33 (0.73–2.39)	0.351
College or Vocational School/Degree Certificate	0.92 (0.48–1.77)	0.804	0.80 (0.45–1.43)	0.451
Graduate School	0.97 (0.50–1.87)	0.918	0.29 (0.16–0.54)	< 0.001*
Insurance (ref: no)				
Yes	1.00 (0.45–2.19)	0.994	0.30 (0.16–0.59)	< 0.001*
Private insurance (ref: no)				
Yes	0.78 (0.57–1.05)	0.102	1.11 (0.80–1.54)	0.550
Medicaid (ref: no)				
Yes	0.69 (0.43–1.11)	0.122	1.27 (0.85–1.91)	0.238
Medicare (ref: no)				
Yes	1.34 (0.97–1.87)	0.080	1.36 (0.97–1.89)	0.073
Disability (ref: no)				
Yes	0.62 (0.46–0.83)	0.002*	1.61 (1.21–2.13)	0.001*
Years since Diagnosis (ref: 0–2 years)				
3–5 years	1.25 (0.88–1.77)	0.222	0.89 (0.62–1.28)	0.533
6–10 years	1.34 (0.95–1.90)	0.094	0.84 (0.59–1.21)	0.355
11 + years	1.09 (0.78–1.53)	0.600	0.70 (0.49–1.00)	0.048*
Place of Residence (ref: urban)				
Suburban	0.74 (0.56–0.96)	0.026*	0.86 (0.64–1.15)	0.304
Rural	0.73 (0.49–1.07)	0.107	1.16 (0.79–1.69)	0.450

*United States, March 2021*

*Significant values are indicated with an asterisk

After adjustment for confounding by other factors, the odds of current tobacco use after cancer diagnosis did not differ significantly based on place of residence. Odds of current tobacco use were significantly lower with increasing age (AOR = 0.97; 95% CI = 0.96–0.98), graduate school education (AOR = 0.29; 95% CI = 0.16–0.54), having insurance (AOR = 0.30; 95% CI = 0.16–0.59), and 11 + years since cancer diagnosis (versus 0–2 years) (AOR = 0.70; 95% CI = 0.49–1.00), and were significantly higher among Hispanic (verses non-Hispanic) (AOR = 1.77; 95% CI = 1.04–3.00) and Black/African American (versus White) (AOR = 2.55; 95% CI = 1.36–4.79) participants.

## Discussion

The goal of this analysis was to assess the association between urbanicity/rurality and both tobacco use and heavy drinking among SGM cancer survivors. Approximately 15% of our sample reported current tobacco use, which is in the low end of rates reported in population health surveillance studies of SGM cancer survivors (12–41%; higher rates reported by bisexual women, specifically) [12, 58–60]. We did not find an association between urbanicity/rurality and tobacco use among SGM cancer survivors, suggesting that prevalence differences were only explained by sociodemographic characteristics and economic determinants. This null finding may also be explained by the sample characteristics – primarily college educated, white males, with health insurance. Approximately 16% of our sample reported heavy drinking, which is higher than rates reported in population health surveillance applying similar measures (5.8–11.5%) [58]. Even controlling for sociodemographic characteristics and economic determinants, we found that SGM cancer survivors living in suburban areas had significantly lower odds of current alcohol use.

Our study suggests that SGM adults are engaged in tobacco use and heavy drinking during cancer survivorship, and that there are disparities in heavy drinking by urbanicity/rurality. These factors could have significant implications on the future health of SGM cancer survivors, such as an increased risk of cancer recurrence or susceptibility to new diseases. Being diagnosed with a life-threatening disease may be a critical period for changing health behaviors and overcoming addiction. This is particularly important for disparate groups such as rural residents and SGM people. Unfortunately, our healthcare system continues to struggle to support cancer survivors to successfully quit alcohol or tobacco use in survivorship. Survivorship Care Plans were not shown to be effective in improving outcomes [61, 62]. More work in research and policy is needed to improve the health of diverse cancer survivors.

Our findings offer unique insight into the use of alcohol and tobacco use among SGM cancer survivors with intersectional, marginalized identities. Participants reporting disability had lower relative odds of current tobacco use, but higher relative odds of heavy drinking. In the general population, a study using 2020 U.S. National Alcohol Survey data, individuals reporting at least one disability had reduced odds of current drinking but greater odds of nicotine use, potentially to self-medicate chronic pain [63]. It is unclear what is driving different results in our SGM sample, though evidence has shown a higher prevalence of disabilities in rural counties as well as in SGM cancer survivors (compared to cisgender, heterosexual survivors), with gender diverse survivors having a disproportionate burden [64, 65] Future research could investigate these combinations of health risk factors with larger, diverse samples.

Furthermore, after receiving a cancer diagnosis, many people continue high-risk health behaviors such as tobacco and alcohol use despite continued risks to their health. In an analysis examining smoking behaviors in approximately 21,000 cancer survivors, 34% quit at diagnosis, and 31% quit after starting cancer treatment; of these, 13% resumed smoking after treatment ended [66]. Patients with smoking-related cancers were more likely to report abstinence. Another study found that cancer survivors who smoked e-cigarettes before diagnosis were four times as likely to continue smoking [67]. With respect to alcohol use, among 34,000 cancer survivors surveyed in the National Health Interview Survey, 56.5% were current drinkers [68]. Of these, 34.9% reported more than moderate or heavy drinking and 21.0% engaged in binge drinking. Examining motivations and behaviors related to urbanicity/rurality could be pertinent to understanding continued alcohol and tobacco usage after cancer diagnosis.

When considering race as a potential confounder, we found that Black/African American participants had greater relative odds of both heavy drinking and tobacco use. Greater alcohol use and tobacco use among Black/African American SGM cancer survivors may result from experiencing, intersectional stressors associated with holding minoritized racial and SGM identities [69]. Black/African American SGM people experience multidimensional structural and interpersonal racism from society at large and from within SGM communities [70–72], which negatively effects health, including substance use [73].

It is important to note that the OUT Study was completed during the COVID-19 pandemic, which could influence rates of alcohol and tobacco use. In a cross-sectional study of 2170 individuals from October 2020 and January 2021 in Brazil, the number of individuals who reported alcohol consumption and tobacco smoking were 9.3% and 14.2%, respectively. The rates of alcohol consumption and smoking increased during COVID-19 to about 20% and 30%, respectively [74]. In subpopulations of SGM individuals, studies have found an increase in nicotine and alcohol usage that is associated with the stress related to the pandemic and SGM identity [75, 76]. There is evidence for severe effects of social isolation on mental health and substance use in SGM people during COVID-19 [20, 77, 78].

### Strengths & Limitations

The OUT Study is one of the few surveys focused on SGM cancer survivors, which is of importance given the high risk for cancer among SGM subpopulations and noted sexual orientation and gender identity-based disparities in social drivers of health (versus the population-at-large). However, there are several limitations that could be addressed in future research. Participants self-reported urbanicity/rurality as urban, suburban, or rural/remote and this may differ substantially from commonly used definitions such as those based on the classifications of residential county or census tract [79]. The spatial patterning of SGM people is likely complex and more nuanced than that reflected by urban/suburban/rural residence, and it was not possible to additionally consider regional, state, city and/or neighborhood-level differences, each of which may impact alcohol and tobacco use through policies, community cohesion, socioeconomic deprivation, race/ethnicity composition, and migration [41–43].

Privacy data protections prevented us from verifying census tracts or county residences in association with commonly used rural/urban classification schemes. It was also not possible to examine potential confounding by spatial patterning. Differences in SGM people’s health behaviors by perceived area of residence vs. rural/urban classifications and spatial patterning could be confirmed in future studies. Further, self-reported cancer site/type, disability and years since diagnosis could not be verified clinically and, therefore, may not accurately reflect the clinical experience of participants; further, additional clinical information, such as specific comorbidities, could not be examined. The participants were cancer survivors and their likelihood of survival may have been associated with risk behavior; those with more alcohol and/or tobacco use may not have survived long enough to participate in the study. We also have no measure of how many participants quit alcohol or tobacco use since being diagnosed with cancer (*e.g.,* former users).

Due to small sub-sample sizes, we collapsed racial/ethnic, gender, and sexual orientation categories. This limitation reduces the statistical power of our analysis when applying these results to large diverse populations. Further, classifying text write-ins for these items was challenging due to the variability and uniqueness of responses. Most participants were of high income, with health insurance, and white, limiting the diversity and generalizability of the sample. Additionally, 44.2% of surveys were incomplete; after excluding these, 2,371 of 2,519 participants (94.1%) were included in the present study; this resulted in smaller analytic samples for data analyses. Lastly, most people who described themselves as gay and lesbian reported being male and female, respectively; therefore, gender effects so strongly aligned with sexual orientation such that distinctions between these factors were not clearly observable.

## Conclusion

Our findings indicate the need for effective interventions to address alcohol and tobacco use among SGM cancer survivors. This may be accomplished by tailoring existing interventions or development of new culturally relevant interventions co-created by the SGM survivor community. Given that alcohol use was greater among SGM cancer survivors living in urban contexts, targeted interventions in urban settings are needed. However, for SGM cancer survivors living in rural/remote areas, increased focus on reducing tobacco use is critical for improving treatment outcomes, reducing risk of recurrence and comorbidities. Interventions that increase screening and referral to supportive services for tobacco and alcohol use in the cancer and primary care settings are one strategy for reaching SGM cancer survivors. However, community-engaged interventions created and implemented with local organizations, including public education campaigns, may be needed to reach SGM cancer survivors beyond the healthcare system.

## Supplementary Information

Below is the link to the electronic supplementary material.

Supplementary file1 (DOCX 32 KB)

## Data Availability

Data set used in this study was provided through a data sharing and use agreement with the National LGBT Cancer Network. Inquiries about the data set are directed toward the National LGBT Cancer Network ([info@cancer-network.org](mailto:info@cancer-network.org)).
