# Supplementary material for: Alcohol and tobacco use among sexual and gender minority cancer survivors in relation to urbanicity/rurality
Source: Cancer Causes Control. 2025 Sep 9;36(12):1869–80. doi: 10.1007/s10552-025-02065-5 (PMC12630166; doi:10.1007/s10552-025-02065-5)
Supplement: Supplementary file 1 — Supplementary file1 (DOCX 32 KB) [file 10552_2025_2065_MOESM1_ESM.docx]

| **Supplemental Table 1. Study sample characteristics and associations with heavy alcohol use & tobacco use among sexual and gender minority cancer survivors included in the final adjusted multivariable model** | | | | |
| --- | --- | --- | --- | --- |
| **Variable Characteristic** | **Alcohol Use (≥2 drinks per day)**  ***N* (Column %)** | | **Tobacco Use**  ***N* (Column %)** | |
|  | **No** | **Yes** | **No** | **Yes** |
| **Age in years (mean SD)** |  |  |  |  |
|  | 58.3 (11.0) | 59.8 (10.7) | 59.1 (10.6) | 55.2 (11.9) |
| **Gender Identity** |  |  |  |  |
| Male | 1015 (79.9%) | 255 (20.1%) | 1044 (82.9%) | 215 (17.1%) |
| Female | 643 (92.3%) | 54 (7.7%) | 617 (89.0%) | 76 (11.0%) |
| Transgender, Genderqueer, Gender non-conforming, Non-Binary, Additional Gender Identities | 108 (92.3%) | 9 (7.7%) | 93 (82.3%) | 20 (17.7%) |
| **Sex assigned at birth** |  |  |  |  |
| Male | 1034 (80.1%) | 257 (19.9%) | 1059 (82.9%) | 218 (17.1%) |
| Female | 732 (92.3%) | 61 (7.7%) | 695 (88.2%) | 93 (11.8%) |
| **Sexual Orientation** |  |  |  |  |
| Gay | 923 (79.3%) | 241 (20.7%) | 964 (83.6%) | 189 (16.4%) |
| Lesbian | 474 (92.2%) | 40 (7.8%) | 455 (88.9%) | 57 (11.1%) |
| Bisexual, Pansexual | 129 (90.8%) | 13 (9.2%) | 117 (83.0%) | 24 (17.0%) |
| Queer | 44 (97.8%) | 1 (2.2%) | 39 (88.6%) | 5 (11.4%) |
| Asexual, Straight, Multiple Orientations--- Additional Orientations | 196 (89.5%) | 283 (10.5%) | 179 (83.3%) | 36 (16.7%) |
| **Race** |  |  |  |  |
| White | 1560 (85.2%) | 271 (14.8%) | 1553 (85.5%) | 263 (14.5%) |
| Black/African American | 44 (68.8%) | 20 (31.2%) | 39 (65.0%) | 21 (35.0%) |
| Indigenous and non-black/African American people of color | 30 (90.9%) | 3 (9.1%) | 29 (85.3%) | 5 (14.7%) |
| Biracial/multiracial | 85 (84.2%) | 16 (15.8%) | 87 (87.9%) | 12 (12.1%) |
| Unclassifiable/Prefer Not to Answer | 47 (85.5%) | 8 (14.5%) | 46 (82.1%) | 10 (17.9%) |
| **Ethnicity** |  |  |  |  |
| Non-Hispanic | 1667 (85.0%) | 295 (15.0%) | 1665 (85.6%) | 279 (14.4%) |
| Hispanic | 99 (81.1%) | 23 (18.9%) | 89 (73.6%) | 32 (26.4%) |
| **Education** |  |  |  |  |
| Highschool Diploma or less | 68 (84.0%) | 13 (16.0%) | 61 (74.4%) | 21 (25.6%) |
| Some College/Vocational School | 314 (86.5%) | 49 (13.5%) | 256 (71.5%) | 102 (28.5%) |
| College or Vocational School/Degree Certificate | 666 (84.3%) | 124 (15.7%) | 648 (82.7%) | 136 (17.3%) |
| Grad School | 718 (84.5%) | 132 (15.5%) | 789 (93.8%) | 52 (6.2%) |
| **Insurance** |  |  |  |  |
| Yes | 50 (83.3%) | 10 (16.7%) | 34 (57.6%) | 25 (42.4%) |
| No | 1716 (84.8%) | 308 (15.2%) | 1720 (85.7%) | 286 (14.3%) |
| **Private insurance** |  |  |  |  |
| Yes | 713 (83.5%) | 141 (16.5%) | 691 (81.4%) | 158 (18.6%) |
| No | 1053 (85.6%) | 177 (14.4%) | 1063 (87.4%) | 153 (12.6%) |
| **Medicaid** |  |  |  |  |
| Yes | 1555 (84.2%) | 291 (15.8%) | 1577 (86.1%) | 254 (13.9%) |
| No | 211 (88.7%) | 27 (11.3%) | 177 (75.6%) | 57 (24.4%) |
| **Medicare** |  |  |  |  |
| Yes | 1102 (86.4%) | 174 (13.6%) | 1083 (85.1%) | 190 (14.9%) |
| No | 664 (82.2%) | 144 (17.8%) | 671 (84.7%) | 121 (15.3%) |
| **Disability** |  |  |  |  |
| Yes | 1116 (82.5%) | 237 (17.5%) | 1182 (87.6%) | 168 (12.4%) |
| No | 650 (88.9%) | 81 (11.1%) | 572 (80.0%) | 143 (20.0%) |
| **Years since Diagnosis** |  |  |  |  |
| 0-2 years | 577 (85.9%) | 95 (14.1%) | 543 (81.8%) | 121 (18.2%) |
| 3-5 years | 348 (83.7%) | 68 (16.3%) | 349 (84.3%) | 65 (15.7%) |
| 6-10 years | 360 (82.9%) | 74 (17.1%) | 370 (86.0%) | 60 (14.0%) |
| 11+ years | 481 (85.6%) | 96 (14.4%) | 492 (88.3%) | 65 (11.7%) |
| **Place of Residence** |  |  |  |  |
| Urban | 647 (81.4%) | 148 (18.6%) | 665 (84.3%) | 124 (15.7%) |
| Suburban | 835 (86.6%) | 129 (13.4%) | 822 (86.3%) | 131 (13.7%) |
| Rural | 284 (87.4%) | 41 (12.6%) | 267 (82.7%) | 56 (17.3%) |
|  |  |  | ***United States, March 2021*** | |
